# Terminal Groups-Dependent Near-Field Enhancement Effect of Ti_3_C_2_T_x_ Nanosheets

**DOI:** 10.1186/s11671-021-03510-5

**Published:** 2021-04-12

**Authors:** Ying-Ying Yang, Wen-Tao Zhou, Wei-Long Song, Qing-Quan Zhu, Hao-Jiang Xiong, Yu Zhang, Sheng Cheng, Pai-Feng Luo, Ying-Wei Lu

**Affiliations:** 1 School of Materials Science and Engineering, Hefei University of Technology, Hefei, 230009 People’s Republic of China; 2 Instrumental Analysis Center, Hefei University of Technology, Hefei, 230009 People’s Republic of China; 3 Engineering Research Center of High Performance Copper Alloy Materials and Processing, Ministry of Education, Hefei University of Technology, Hefei, 230009 People’s Republic of China

**Keywords:** Multilayered Ti_3_C_2_T_x_, Few-layered Ti_3_C_2_T_x_, Terminal group, Near-field enhancement, Coupling

## Abstract

Both multilayered (ML) and few-layered (FL) Ti_3_C_2_T_x_ nanosheets have been prepared through a typical etching and delaminating procedure. Various characterizations confirm that the dominant terminal groups on ML-Ti_3_C_2_T_x_ and FL-Ti_3_C_2_T_x_ are different, which have been assigned to O-related and hydroxyl groups, respectively. Such deviation of the dominant terminals results in the different physical and chemical performance and eventually makes the nanosheets have different potential applications. In particular, before coupling to Ag nanoparticles, ML-Ti_3_C_2_T_x_ can present stronger near-field enhancement effect; however, Ag/FL-Ti_3_C_2_T_x_ hybrid structure can confine stronger near-field due to the electron injection, which can be offered by the terminated hydroxyl groups.

**Supplementary Information:**

The online version contains supplementary material available at 10.1186/s11671-021-03510-5.

## Introduction

Ti_3_C_2_T_x_, a typical two-dimensional layered transition metal carbide with a graphene-like structure, has attracted great attention due to its wide potential applications in fields of catalysis, energy, and medicine thanks to its unique properties, especially large specific surface area and so on [1–6]. It has been demonstrated that the physical and chemical performance of Ti_3_C_2_T_x_ could be determined by its terminal groups, referred as T_x_ in the formula (usually are –F, –O and/or –OH), which can be adjusted by choosing different preparation procedures [7, 8]. For example, some experimental results indicate that the hydrophilic hydrophobic equilibrium of Ti_3_C_2_T_x_ can be modulated by interacting some agent groups with –O terminal groups on Ti_3_C_2_T_x_ [9], and the Pb adsorption capacity can be improved by connecting with hydroxyl groups on Ti_3_C_2_T_x_ [10]. In the meantime, some theoretical works have determined that the attached methoxy groups could improve the stability of Ti_2_C and Ti_3_C_2_ [11], and O-related terminal groups could enhance the lithium ion storage capacity of various nanosheets [12]. Apart from the multifarious applications by taking advantage of the unique layered structure with certain terminal groups, it is found that Ti_3_C_2_T_x_ can present plasmonic performance as well, and the resonance wavelength can be tuned by the terminals and/or thickness [13], indicating that Ti_3_C_2_T_x_ could confine electromagnetic field under excitation and eventually can be employed as broadband perfect absorbers [14, 15], Terahertz shielding devices [16], and photonic and/or molecular detectors or sensors [17–19]. However, most of previous works either concerned the etching condition dependent terminal groups [20] or focused on the overall plasmonic performance [21]. Therefore, it is interesting to systematically study the relationship between the terminal groups of Ti_3_C_2_T_x_ with different layers and their near-field enhancement effect, since such effect has been widely employed in many optical related fields, such as surface-enhanced Raman scattering detection, due to the strong confined electromagnetic field [22–24].

In this work, in order to simplify the terminal options and avoid using hazardous HF, the mixed etching agent of LiF and HCl has been used to minimize the fluorine terminals (–F) in the etching process [25]. Furthermore, the procedure of sonication in water has been carried out to delaminate the multilayered Ti_3_C_2_T_x_ (ML-Ti_3_C_2_T_x_) into few-layered Ti_3_C_2_T_x_ (FL-Ti_3_C_2_T_x_) without introducing any other reagents. As a result, the obtained Ti_3_C_2_T_x_ with different layers in this work will be mainly terminated by either O- or OH-related groups, which make ML-Ti_3_C_2_T_x_ or FL-Ti_3_C_2_T_x_ nanosheets reveal different physical and chemical properties and eventually present different near-filed enhancement performance**.** In addition, the hybrid structures composed of Ti_3_C_2_T_x_ and Ag nanoparticles have been prepared and the corresponding coupling effects have been explored as well. Such exploration regarding terminal dependent plasmonic performance of these Ti_3_C_2_T_x_ with different layers and configurations could help people to select suitable Ti_3_C_2_T_x_-based materials in some specific optical fields.

## Methods

### Preparation of Ti_3_C_2_T_x_ Nanosheets

ML-Ti_3_C_2_T_x_ was prepared by following a modified previously reported method [26]. The typical etching process started with the preparation of LiF solution by dissolving 1 g of LiF in 20 mL of dilute HCl solution (6 M) with stirring. Subsequently, 1 g of Ti_3_AlC_2_ powder was slowly added into the above solution, and the etching process was kept at 70 °C for 45 h under stirring. The wet sediment was then washed several times with deionized water until the pH of the suspension liquid was bigger than 6. Afterward, the suspension was collected and named as ML-Ti_3_C_2_T_x_. To obtain FL-Ti_3_C_2_T_x_, ML-Ti_3_C_2_T_x_ was further delaminated by sonication for 2 h in Ar atmosphere and followed by centrifugation at 3500 rpm for 1 h.

### Preparation of Ag/Ti_3_C_2_T_x_ Nanocomposites

The synthesis of the hybrid materials was started with the preparation of the mixed solution of AgNO_3_ (12.5 mL, 2 mmol/L) and NaC_6_H_5_O_7_ (12.5 mL, 4 mmol/L) at room temperature. After rapidly adding PVP solution (25 mL, 0.1 g/mL), Ti_3_C_2_T_x_ solution (5 mL, 0.05 mg/mL) was then slowly added into the mixed solution with stirring for 10 min at room temperature. Subsequently, the above-mixed solution was heated up to 70 °C to react for 45 h. After centrifuging, the products were kept in water and named as Ag/ML-Ti_3_C_2_T_x_ and Ag/FL-Ti_3_C_2_T_x_, respectively, according to the type of Ti_3_C_2_T_x_ used in the procedure.

### Characterization

A field emission scanning electron microscope (Carl ZEISS Sigma) and two transmission electron microscopes (JEM-2100F and JEM-1400Flash) have been employed to determine the morphologies of the samples. The X-ray diffraction (XRD) patterns in the range of 2θ = 5°–80° with a step of 0.02° were recorded on a powder diffractometer (X'Pert PRO MPD). Zeta potentials and surface states of ML-Ti_3_C_2_T_x_ and FL-Ti_3_C_2_T_x_ were measured by a Malvern Zetasizer (Nano-ZS90) and an X-ray photoelectron spectroscopy (XPS, ESCALAB 250Xi), respectively. The absorption and Raman performance of samples were recorded by a UV–Vis spectrophotometer (CARY 5000) and a Raman spectroscopy (LabRAM HR Evolution), respectively. The excitation wavelength of Raman detection was 532 nm, and the laser powers for usual Raman measurements and surface enhanced Raman scattering (SERS) characterizations were 12.5 mW and 0.05 mW, respectively.

## Results and Discussion

**Fig. 1:**
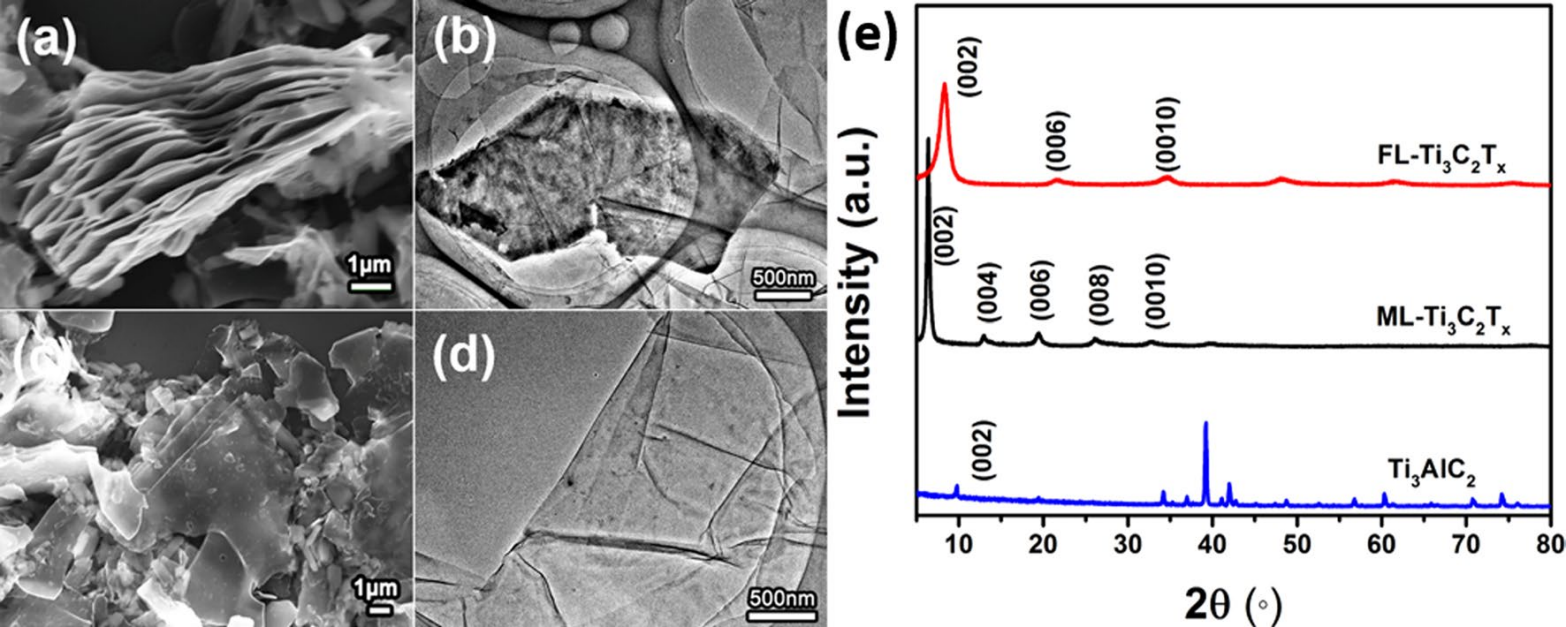
Morphology and phase determinations. **a**, **b** SEM and TEM images of ML-Ti_3_C_2_T_x_. **c**, **d** SEM and TEM images of FL-Ti_3_C_2_T_x_. **e** XRD patterns of Ti_3_AlC_2_, ML-Ti_3_C_2_T_x_ and FL-Ti_3_C_2_T_x_

Both morphologies of ML-Ti_3_C_2_T_x_ and FL-Ti_3_C_2_T_x_ are shown in Fig. 1a, b and c, d, respectively. It can be seen that FL-Ti_3_C_2_T_x_ looks more transparent, indicating that its layer number is much less than ML-Ti_3_C_2_T_x_. Figure 1e shows the XRD patterns of all samples. Ti_3_AlC_2_ and ML-Ti_3_C_2_T_x_ show their typical phase features, which agree well with some previous reports [26–28]_._ It can be readily observed that the intense (002) peak of ML-Ti_3_C_2_T_x_ shifts to the lower angle comparing with that of Ti_3_AlC_2_, implying the removal of Al atoms from the MAX phase and the expanding along the c axis. Compared with the diffraction peaks of ML-Ti_3_C_2_T_x_, both broadened (002) peak and disappeared (004) and (008) peaks of FL-Ti_3_C_2_T_x_ determined the successful preparation of the few-layered sample [29]. Moreover, the (002) peak of FL-Ti_3_C_2_T_x_ locates at a little higher angle than that of ML-Ti_3_C_2_T_x_, indicating that ML-Ti_3_C_2_T_x_ and FL-Ti_3_C_2_T_x_ should be terminated with different groups, which can be attributed to -O and -OH, respectively, since the as-prepared Ti_3_C_2_T_x_ (ML-Ti_3_C_2_T_x_) will not be mainly terminated with -F without HF as etching agent and the corresponding c parameters attracted from the XRD patterns agree well with what previous works reported [25, 30].

**Fig. 2:**
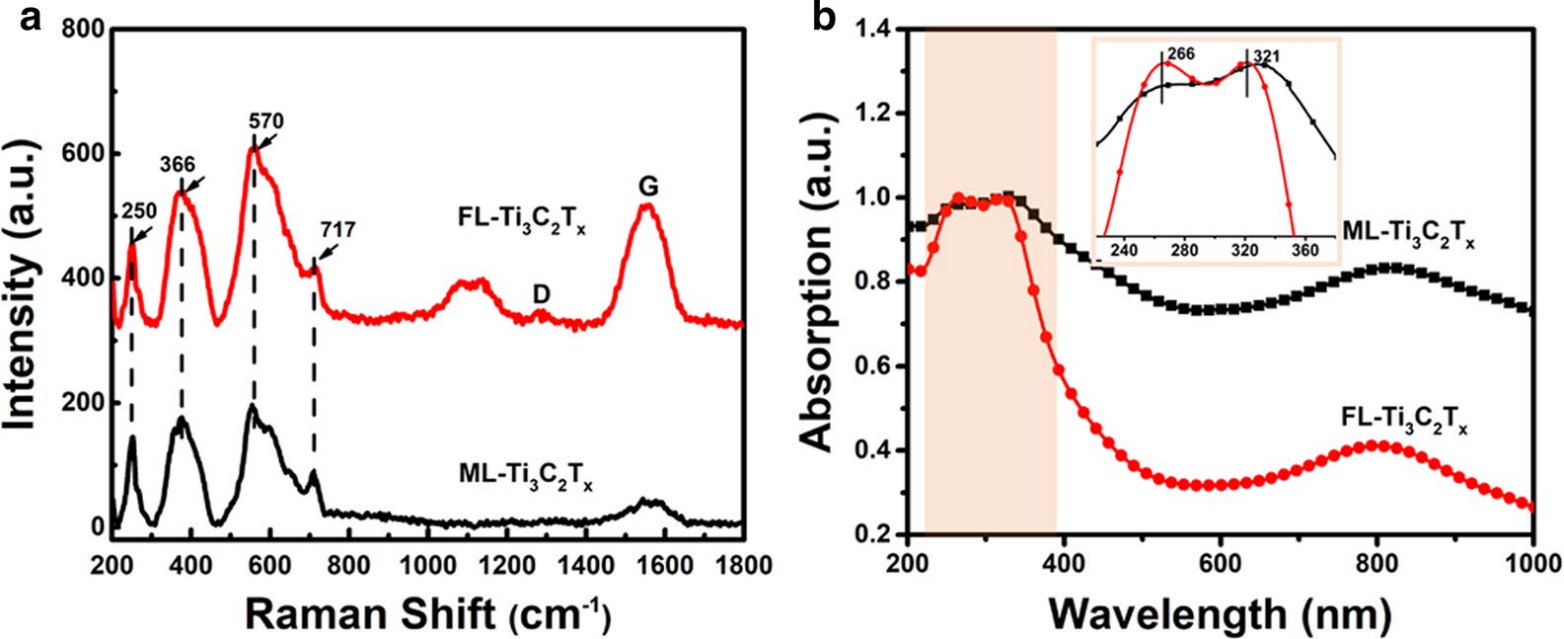
**a** Raman spectra and **b** Normalized absorption spectra of FL-Ti_3_C_2_T_x_ and ML-Ti_3_C_2_T_x_. The inset in **b** presents the absorption bands of FL-Ti_3_C_2_T_x_ and ML-Ti_3_C_2_T_x_ in the UV region

Figure 2a shows Raman spectra of ML-Ti_3_C_2_T_x_ and FL-Ti_3_C_2_T_x_. As it can be seen that the Raman signals in the range of 200–800 cm^−1^ for both samples are quite similar. Among them, the peak at 717 cm^−1^ is due to the A_1g_ symmetrical out-of-plane vibration of Ti and C atoms, while the peaks at 244, 366 and 570 cm^−1^ are arising from the in-plane (shear) modes of Ti, C and surface terminal groups, respectively [31, 32]. As for the Raman signals ranging from 800 to 1800 cm^−1^, comparing with ML-Ti_3_C_2_T_x_, FL-Ti_3_C_2_T_x_ not only shows stronger Raman signal at 1580 cm^−1^ (G band), but also presents two emerging Raman bands at 1000–1200 cm^−1^ and 1300 cm^−1^ (D band). Herein, the appearance of D band indicates that some Ti atoms have been peeled away and more C atoms are exposed to the surroundings [33]. Therefore, the integrated Raman intensity of FL-Ti_3_C_2_T_x_ in this range is slightly larger than that of ML-Ti_3_C_2_T_x_, implying that FL-Ti_3_C_2_T_x_ adsorbs more terminal groups. Zeta potentials of ML-Ti_3_C_2_T_x_ and FL-Ti_3_C_2_T_x_ are −4.38 and −26.9 mV, respectively, as shown in Additional file 1: Fig. S1, which further confirm that FL-Ti_3_C_2_T_x_ are terminated by more groups with negative charges.

The UV–Vis spectra shown in Fig. 2b reveal that both FL-Ti_3_C_2_T_x_ and ML-Ti_3_C_2_T_x_ present two dominant absorption bands. In the UV region (225–325 nm), FL-Ti_3_C_2_T_x_ displays relatively stronger absorption band which corresponds to the band gap transition [34], implying that there are more -OH groups have been terminated on FL-Ti_3_C_2_T_x_ [35]. On the other hand, the comparison between the long wavelength absorption bands (600-1000 nm) of both samples shows that the relative intensity of FL-Ti_3_C_2_T_x_ in this range is obviously lower than that of ML-Ti_3_C_2_T_x_, indicating that ML-Ti_3_C_2_T_x_ are mainly terminated by –O [35]. FL-Ti_3_C_2_T_x_ can be well dispersed in the aqueous solution since the terminated –OH groups shows hydrophilicity and electrostatic repulsion between sheets [31, 36]. As for ML-Ti_3_C_2_T_x_ with more –O terminals, it can only form a suspension in the beginning and will deposit subsequently as shown in Additional file 1: Fig. S2a.

**Fig. 3:**
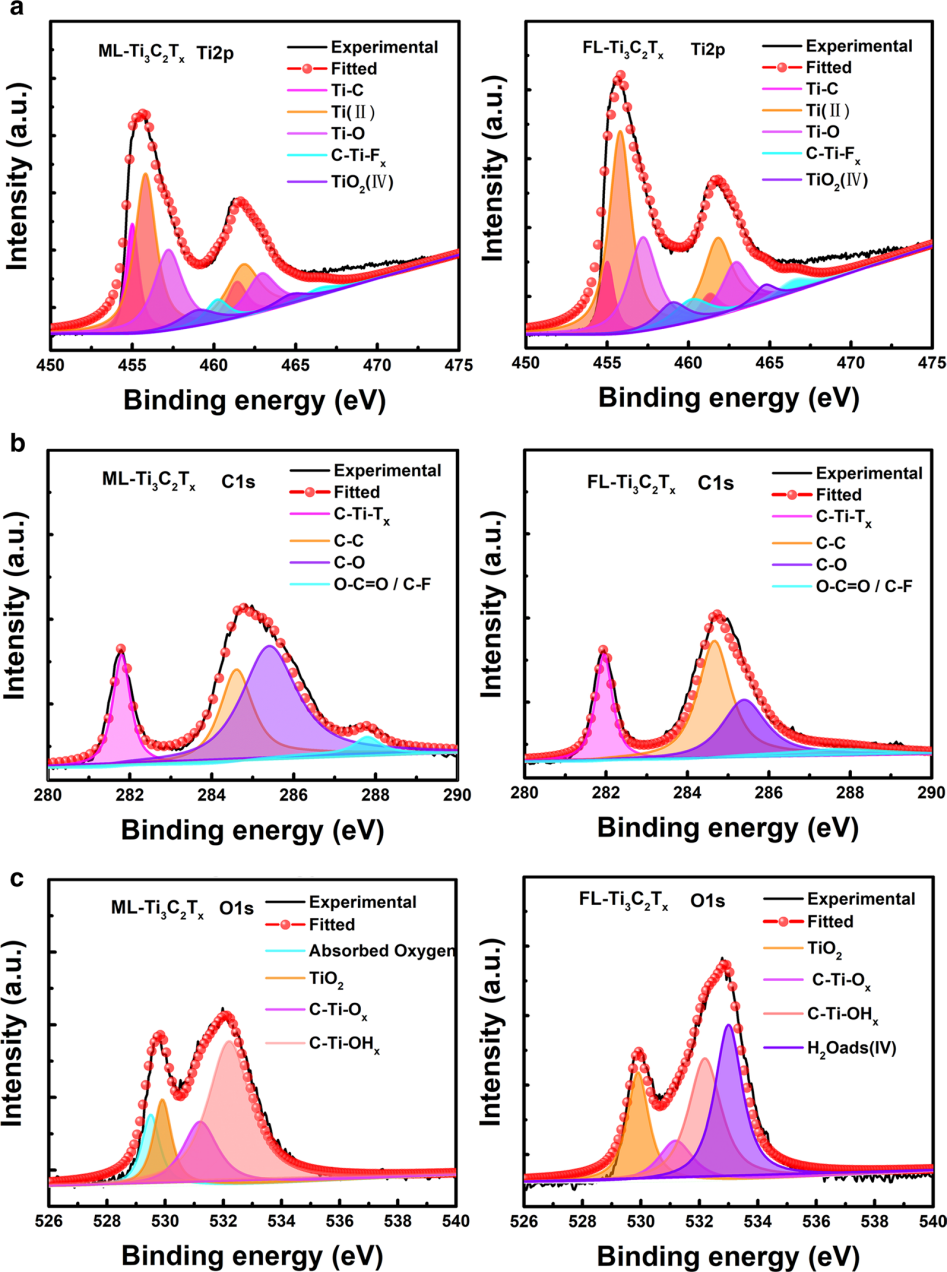
XPS spectra of ML-Ti_3_C_2_T_x_ and FL-Ti_3_C_2_T_x_ **a** Ti2p, **b** C1s, **c** O1s

In order to shed more light on the surface groups terminated on ML-Ti_3_C_2_T_x_ and FL-Ti_3_C_2_T_x_, XPS spectra of both samples were collected and are shown in Fig. 3. All corresponding detailed information regarding the surface states are summarized in Additional file 1: Table S1. The fraction of Ti-C in FL-Ti_3_C_2_T_x_ (9.80%) is lower than that in ML-Ti_3_C_2_T_x_ (17.31%), while the ratio of C–C in FL-Ti_3_C_2_T_x_ (44.62%) is higher. Such surface states changing evidences the loss of Ti atoms and the more exposed C atoms on the surface of FL-Ti_3_C_2_T_x_, which agrees with the emerging D band in its Raman spectrum shown in Fig. 2a. The increased C-Ti-T_x_ ratio in FL-Ti_3_C_2_T_x_ (21.27%) indicates that there should be more active terminal groups adsorbed on its surface than ML-Ti_3_C_2_T_x_, which agrees with the Zeta potential results shown in Additional file 1: Fig. S1. Apart from the quantity of the terminal groups, the analysis of XPS results also reveals that FL-Ti_3_C_2_T_x_ and ML-Ti_3_C_2_T_x_ have been terminated by different dominant functional groups, which also has been suggested by the (002) diffraction peaks shown in Fig. 1e. Regarding O 1 s spectra of these two samples, it can be clearly seen that more O-related states have been found on the surface of ML-Ti_3_C_2_T_x_, and some of them are adsorbed oxygen molecules, which can dissociate to form Ti_3_C_2_O_x_ and therefore will repel O_2_ in air to prevent further oxidation of ML-Ti_3_C_2_T_x_ [37]. As a result, ML-Ti_3_C_2_T_x_ seems present a better oxidation resistance with a lower TiO_2_ ratio (13.98%) than FL-Ti_3_C_2_T_x_ (19.60%).

Based on the observations and analyses of Figs. 1, 2 and 3, it can be concluded that although both ML-Ti_3_C_2_T_x_ and FL-Ti_3_C_2_T_x_ are terminated by some functional groups with negative charge, the amount and dominant type of the groups are quite different. On one hand, the quantity of terminal groups on FL-Ti_3_C_2_T_x_ is larger than that of ML-Ti_3_C_2_T_x_. On the other hand, the dominant terminal structure on ML-Ti_3_C_2_T_x_ is Ti_3_C_2_O_2_, which makes ML-Ti_3_C_2_T_x_ to be more stable in the air [38], while for FL-Ti_3_C_2_T_x_, it is mainly terminated by Ti_3_C_2_(OH)_2_, which helps FL-Ti_3_C_2_T_x_ to be well-dispersed in aqueous solutions [36].

**Fig. 4:**
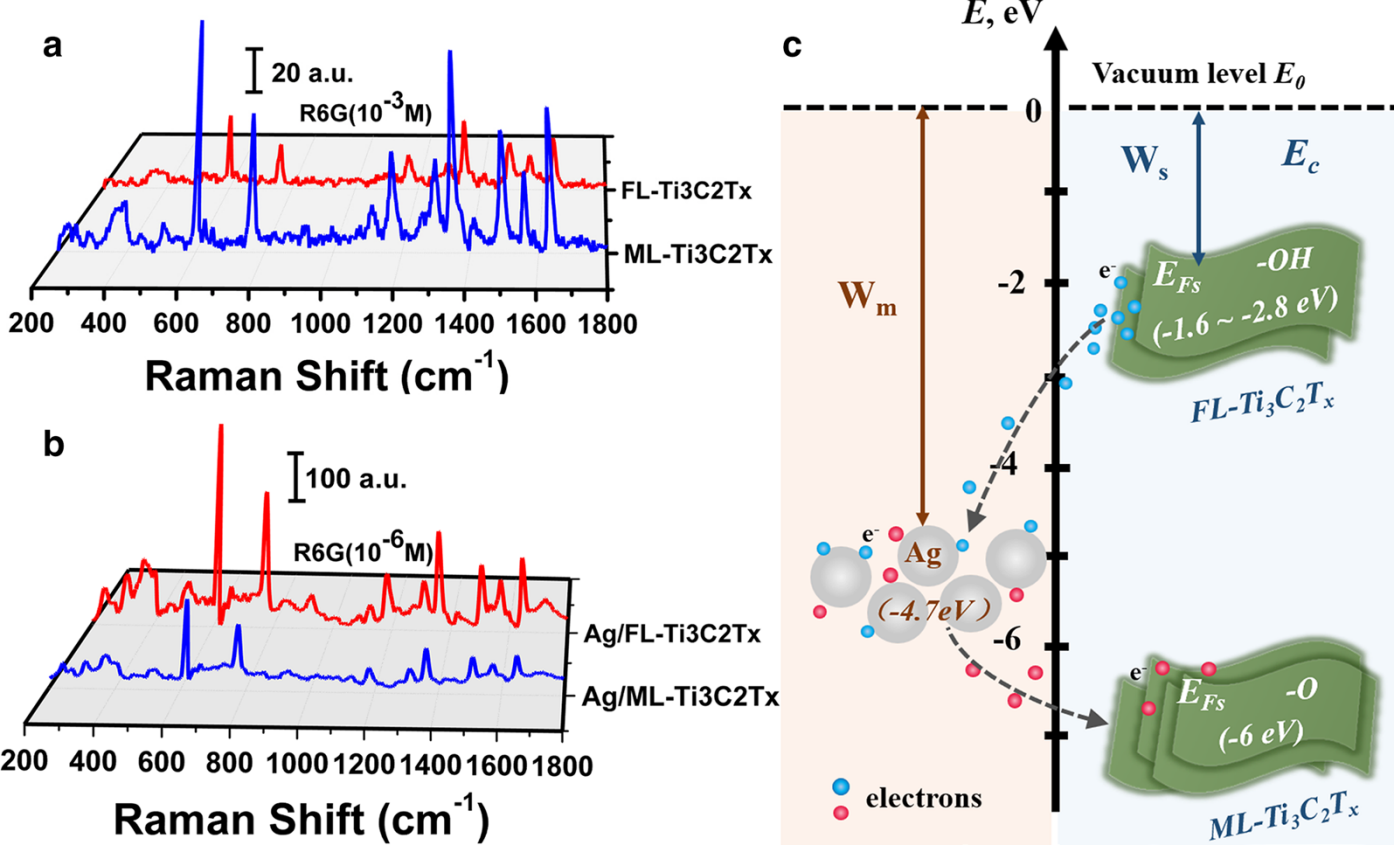
**a** SERS spectra of R6G (10^–3^ M) with ML-Ti_3_C_2_T_x_ and FL-Ti_3_C_2_T_x_. **b** SERS spectra of R6G (10^–6^ M) with Ag/ML-Ti_3_C_2_T_x_ and Ag/FL-Ti_3_C_2_T_x_. **c** Schematic diagram of electron transfer from FL-Ti_3_C_2_T_x_ to Ag NP due to their work function difference. W_m_ and W_s_ represent the work functions of Ag NP and FL-Ti_3_C_2_T_x_, respectively

Ti_3_C_2_T_x_ with functional terminal groups could reveal good adsorption performance and therefore could act as a surface-enhanced Raman scattering (SERS) substrate to improve the Raman activity of positively charged probe molecules [3, 39, 40]. Comparing with ML-Ti_3_C_2_T_x_, FL-Ti_3_C_2_T_x_ should present better adsorption ability since it has been determined that it is terminated with more negative charges. Such better adsorption performance has been demonstrated by the optical photographs of the mixed solution with R6G and FL-Ti_3_C_2_T_x_ as shown in Additional file 1: Fig. S2b. However, Fig. 4a reveals that the ML-Ti_3_C_2_T_x_ substrate obviously performs better SERS activity than FL-Ti_3_C_2_T_x_ one. Considering ML-Ti_3_C_2_T_x_ with –O terminal presents stronger absorption band centered at around 800 nm, which can be assigned to the surface plasmon resonant absorption [3, 15, 39, 41], it therefore can be concluded that ML-Ti_3_C_2_T_x_ with stronger SERS activity should result from the stronger near-field effect induced by the relatively stronger surface plasmon resonance as shown in Fig. 2b.

In order to further explore the relationship between the terminal groups and the near-filed effect of Ti_3_C_2_T_x_ nanosheets, the hybrid structures composed of Ti_3_C_2_T_x_ nanosheets, including few layered and multilayered, and Ag nanoparticles (NPs) have been synthesized, which are accordingly labeled as Ag/FL-Ti_3_C_2_T_x_ and Ag/ML-Ti_3_C_2_T_x_, respectively. The morphologies of both hybrid samples are shown in Additional file 1: Fig. S3. The insets indicate the corresponding size distributions of Ag NPs loading on ML-Ti_3_C_2_T_x_ (5–40 nm) is larger than that on FL-Ti_3_C_2_T_x_ (2–20 nm). Intuitively, it might be concluded that Ag/ML-Ti_3_C_2_T_x_ could perform better SERS activity than Ag/FL-Ti_3_C_2_T_x_ since both larger Ag NPs and relative stronger surface plasmon resonance of ML-Ti_3_C_2_T_x_ are beneficial to confine stronger near-field. However, the SERS spectra shown in Fig. 4b reveal a counterintuitive result. It is clear that the enhancement effect offered by Ag/FL-Ti_3_C_2_T_x_ is nearly 3 times of that by Ag/ML-Ti_3_C_2_T_x_, implying that the coupling between Ag NPs and FL-Ti_3_C_2_T_x_ should play an important role during the detection process. As confirmed above that FL-Ti_3_C_2_T_x_ has been mainly terminated by -OH groups with lots of surface electrons, which will result in the formation of Ti_3_C_2_(OH)_2_ structure with a work function of 1.6–2.8 eV [42, 43]. As shown in Fig. 4c, the abundant surface electrons will therefore transfer from FL-Ti_3_C_2_T_x_ to Ag NPs with a work function of 4.7 eV [44]. With the extra injection of hot electrons from FL-Ti_3_C_2_T_x_, Ag NPs with smaller size could present stronger resonance under the excitation and eventually perform better SERS activity due to the coupling induced stronger electromagnetic effect. It is worth noting that the work function of Ti_3_C_2_O_2_ structure formed on the surface of ML-Ti_3_C_2_T_x_ is around 6.0 eV [43], which will result in electron transfer from Ag NPs surface to ML-Ti_3_C_2_T_x_ nanosheets and therefore will weaken the near-field enhanced effect supported by the Ag NPs. On the other hand, not like FL-Ti_3_C_2_T_x_ with -OH terminals, ML-Ti_3_C_2_T_x_ with -O terminals cannot offer sufficient electrons under excitation [42]. It is therefore reasonable that the SERS activity of Ag/ML-Ti_3_C_2_T_x_ is worse than that of Ag/ FL-Ti_3_C_2_T_x_.

## Conclusions

In summary, ML-Ti_3_C_2_T_x_ and FL-Ti_3_C_2_T_x_ terminated with different dominant functional groups have been successfully prepared. It has been demonstrated that ML-Ti_3_C_2_T_x_ is more stable in the air due to the surface structure of Ti_3_C_2_O_2_ and show stronger SERS activity than FL-Ti_3_C_2_T_x_ because it can reveal stronger near-field effect. However, FL-Ti_3_C_2_T_x_ terminated by Ti_3_C_2_(OH)_2_ can be well dispersed in aqueous solution and will show better SERS performance after coupling to the Ag NPs due to the sufficient electron injection. Such research regarding the terminal groups-dependent near-field enhancement performance will help people to expand the potential applications of Ti_3_C_2_T_x_ in the optical related fields.

## Supplementary Information

**Additional file 1.** **Figure S1.** Zeta potentials of ML-Ti_3_C_2_T_x_ and FL-Ti_3_C_2_T_x_. **Figure S2.** (a) Optical photographs of ML-Ti_3_C_2_T_x_ and FL-Ti_3_C_2_T_x_. (b) Optical photographs of ML-Ti_3_C_2_T_x_ and FL-Ti_3_C_2_T_x_ soaking in R6G solutions. **Figure S3.** TEM images of (a) Ag/ML-Ti_3_C_2_T_x_ and (b) Ag/FL-Ti_3_C_2_T_x_. The insets are the size distributions of Ag NPs in the corresponding samples. **Table S1.** Surface states and corresponding relative contents extracted from the XPS Ti 2p, C 1s and O 1s spectra of ML-Ti_3_C_2_T_x_ and FL-Ti_3_C_2_T_x_.

## Data Availability

The raw dataset obtained analyzed during the experimental work is avaiable from the corresponding author on reasonable request.

## Abbreviations

ML-Ti_3_C_2_T_x_: Multilayered Ti_3_C_2_T_x_
FL-Ti_3_C_2_T_x_: Few layered Ti_3_C_2_T_x_
SERS: Surface enhanced Raman scattering
NPs: Nanoparticles
