# Supplementary material for: Terminal Groups-Dependent Near-Field Enhancement Effect of Ti3C2Tx Nanosheets
Source: Nanoscale Res Lett. 2021 Apr 12;16:60. doi: 10.1186/s11671-021-03510-5 (PMC8041988; doi:10.1186/s11671-021-03510-5)
Supplement: Supplementary file 1 — Additional file 1. Figure S1. Zeta potentials of ML-Ti3C2Tx and FL-Ti3C2Tx. Figure S2. (a) Optical photographs of ML-Ti3C2Tx and FL-Ti3C2Tx. (b) Optical photographs of ML-Ti3C2Tx and FL-Ti3C2Tx soaking in R6G solutions. Figure S3. TEM images of (a) Ag/ML-Ti3C2Tx and (b) Ag/FL-Ti3C2Tx. The insets are the size distributions of Ag NPs in the corresponding samples. Table S1. Surface states and corresponding relative contents extracted from the XPS Ti 2p, C 1s and O 1s spectra of ML-Ti3C2Tx and FL-Ti3C2Tx. [file 11671_2021_3510_MOESM1_ESM.docx]

# Supporting Information

**Terminal Groups Dependent Near-Field Enhancement Effect of Ti_3_C_2_T_x_ Nanosheets**

Ying-Ying Yang^1^, Wen-Tao Zhou^1^, Wei-Long Song^1^, Qing-Quan Zhu^1^, Hao-Jiang Xiong^1^, Jue Zhang^1^, Sheng Cheng^2^, Pai-Feng Luo^1^, Ying-Wei Lu^1, 3,^ ^[[1]](#footnote-1)^

^1^School of Materials Science and Engineering, Hefei University of Technology, Hefei 230009, P. R. China

^2^Instrumental Analysis Center, Hefei University of Technology, Hefei 230009, P. R. China

^3^Engineering Research Center of High Performance Copper Alloy Materials and Processing, Ministry of Education, Hefei University of Technology, Hefei 230009, P. R. China





Figure S1. Zeta potentials of ML-Ti_3_C_2_T_x_ and FL-Ti_3_C_2_T_x_


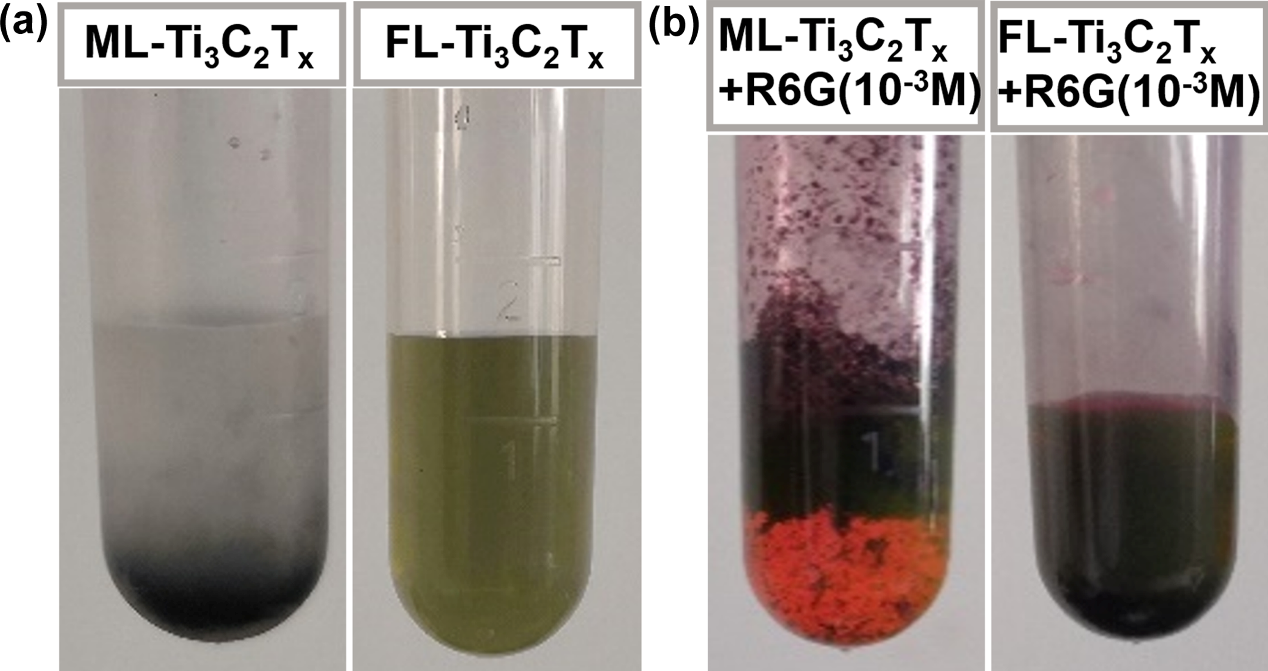


Figure S2. (a) Optical photographs of ML-Ti_3_C_2_T_x_ and FL-Ti_3_C_2_T_x_. (b) Optical photographs of ML-Ti_3_C_2_T_x_ and FL-Ti_3_C_2_T_x_ soaking in R6G solutions.

Table S1. Surface states and corresponding relative contents extracted from the XPS Ti 2p, C 1s and O 1s spectra of ML-Ti_3_C_2_T_x_ and FL-Ti_3_C_2_T_x_

| Sample IDs | | | ML-Ti_3_C_2_T_x_ | FL-Ti_3_C_2_T_x_ |
| --- | --- | --- | --- | --- |
| Region | BE (eV) | Assigned to | Fraction (%) | Fraction (%) |
| Ti 2p* | 455.0/461.2 | Ti-C^1-3^ | 17.31 | 9.80 |
|  | 455.8/461.8 | Ti(II) ^1,3^ | 41.47 | 46.35 |
|  | 457.2/462.9 | Ti-O^1,2^ | 28.10 | 28.02 |
|  | 459.0/464.7 | TiO_2_^1^ | 6.15 | 8.37 |
|  | 460.2/466.5 | C-Ti-F_x_(III) ^1^ | 6.90 | 7.46 |
| C 1s | 281.8 | C-Ti-T_x_^1^ | 17.23 | 21.27 |
|  | 284.6 | C-C^1,2^ | 26.17 | 44.62 |
|  | 285.4 | C-O^1,2^ | 53.00 | 28.58 |
|  | 287.8 | O-C=O/C-F^2,3^ | 3.60 | 5.53 |
| O 1s | 529.4 | Adsorbed Oxygen^4^ | 11.54 | - |
|  | 529.9 | TiO_2_^1^ | 13.98 | 19.60 |
|  | 531.2 | C-Ti-O_x_^1^ | 19.91 | 11.64 |
|  | 532.2 | C-Ti-OH_x_^1,5^ | 54.57 | 33.21 |
|  | 533.1 | H_2_Oads or C-OH^1^ | - | 35.55 |

* XPS Ti 2p spectra presents Ti 2p_3/2_ (lower BE) and Ti 2p_1/2_ (higher BE) components


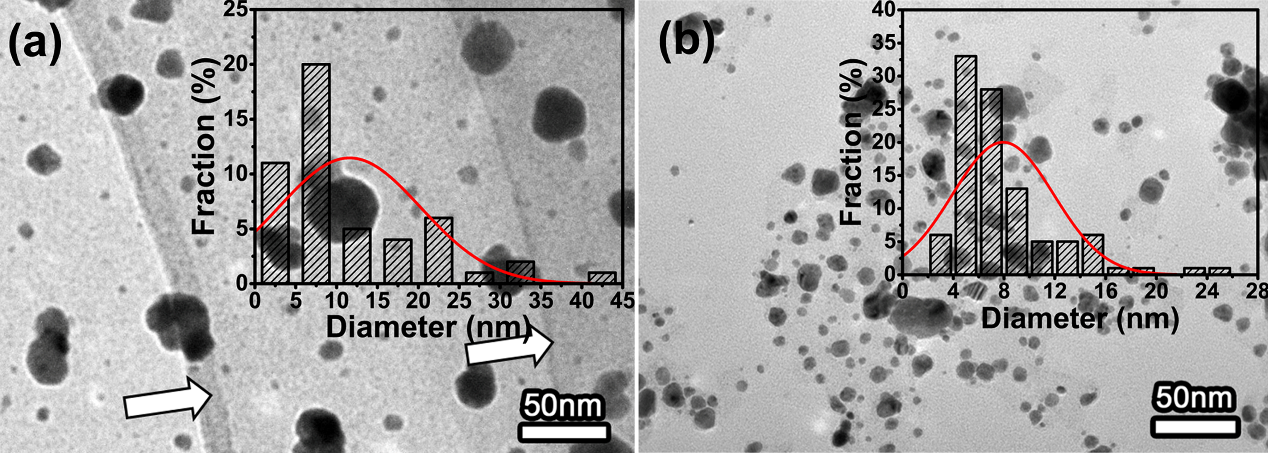


Figure S3. TEM images of (a) Ag/ML-Ti_3_C_2_T_x_ and (b) Ag/FL-Ti_3_C_2_T_x_. The insets are the size distributions of Ag NPs in the corresponding samples.

**References**

1. Halim J, Cook K M, Naguib M, Eklund P, Gogotsi Y, Resen J, Barsoum MW (2016) X-ray photoelectron spectroscopy of select multi-layered transition metal carbides (MXenes). Appl. Surf. Sci. 362: 406-417.
2. Han M, Yin X, Li X, Anasori B, Zhang L, Cheng L, Gogotsi Y (2017) Laminated and two-dimensional carbon-supported microwave absorbers derived from MXenes. ACS Appl. Mater. Interfaces 9: 20038-20045.
3. Tong Y, He M, Zhou Y, Zhong X, Fan L, Huang T, Liao Q, Wang Y (2018) Electromagnetic wave absorption properties in the centimetre-band of  Ti_3_C_2_T_x_ MXenes with diverse etching time. J. Mater. Sci.-Mater. El. 29: 8078-8088.
4. Peng C, Yang X, Li Y, Yu H, Wang H, Peng F (2016) Hybrids of two-dimensional Ti_3_C_2_ and TiO_2_ exposing {001} facets toward enhanced photocatalytic activity. ACS Appl. Mater. Interfaces 8: 6051-6060.
5. Mashtalir O, Naguib M, Mochalin VN, Dall’Agnese Y, Heon M, Barsoum MW, Gogotsi Y (2013) Intercalation and delamination of layered carbides and carbonitrides. Nat.Commum. 4: 1-7.

1. Correspondence: [luyw@hfut.edu.cn](mailto:luyw@hfut.edu.cn)

   ^1^School of Materials Science and Engineering, Hefei University of Technology, Hefei 230009, P. R. China

   ^3^Engineering Research Center of High Performance Copper Alloy Materials and Processing, Ministry of Education, Hefei University of Technology, Hefei 230009, P. R. China [↑](#footnote-ref-1)
